# Disseminated intravascular coagulation with the fibrinolytic phenotype predicts the outcome of patients with out-of-hospital cardiac arrest

**DOI:** 10.1186/s12959-016-0116-y

**Published:** 2016-09-21

**Authors:** Takeshi Wada, Satoshi Gando, Yuichi Ono, Kunihiko Maekawa, Kenichi Katabami, Mineji Hayakawa, Atsushi Sawamura

**Affiliations:** Division of Acute and Critical Care Medicine, Department of Anesthesiology and Critical Care Medicine, Hokkaido University Graduate School of Medicine, N15W7, Kita-ku, Sapporo, 060-8638 Japan

**Keywords:** Cardiac arrest, Disseminated intravascular coagulation (DIC), Fibrinolysis, Outcome, Out-of-hospital

## Abstract

**Background:**

We tested the hypothesis that disseminated intravascular coagulation (DIC) during the early phase of post-cardiopulmonary resuscitation (CPR) is associated with systemic inflammatory response syndrome (SIRS), multiple organ dysfunction syndrome (MODS) and affects the outcome of out-of-hospital cardiac arrest (OHCA) patients.

**Methods:**

A review of the computer-based medical records of OHCA patients was retrospectively conducted and included 388 patients who were divided into DIC and non-DIC patients based on the Japanese Association for Acute Medicine DIC diagnostic criteria. DIC patients were subdivided into two groups: those with and without hyperfibrinolysis. Pre-hospital factors, platelet count, coagulation and fibrinolysis markers and lactate levels within 24 h after resuscitation were evaluated. The outcome measure was all-cause hospital mortality.

**Results:**

DIC patients exhibited lower platelet counts, prolonged prothrombin time, decreased levels of fibrinogen and antithrombin associated with increased fibrinolysis than those without DIC. DIC patients more frequently developed SIRS and MODS, followed by worse outcomes than non-DIC patients. The same changes were observed in DIC patients with hyperfibrinolysis who showed a higher prevalence of MODS, leading to worse outcome than those without hyperfibrinolysis. Logistic regression analyses showed that lactate levels predicted hyperfibrinolysis and DIC is an independent predictor of patient death. Survival probabilities of DIC patients during hospital stay were significantly lower than non-DIC patients. The area under the receiver operating characteristic curve of DIC for the prediction of death was 0.704.

**Conclusions:**

The fibrinolytic phenotype of DIC during the early phase of post-CPR more frequently results in SIRS and MODS, especially in patients with hyperfibrinolysis, and affects the outcome of OHCA patients.

**Electronic supplementary material:**

The online version of this article (doi:10.1186/s12959-016-0116-y) contains supplementary material, which is available to authorized users.

## Background

Whole body ischemia reperfusion due to cardiac arrest constitutes post-resuscitation syndrome resulting from microvascular obstruction-induced tissue hypoxia in many vital organs, which affects the patient’s outcome [1]. Microvascular obstruction, referred to as the no reflow phenomenon, in the brain has been attributed to intravascular thrombosis during cardiac arrest [1, 2]. Post-resuscitation syndrome is now referred to as post-cardiac arrest syndrome consisting of four syndromes including systemic ischemia reperfusion responses and post-cardiac arrest brain injury [3]. Main pathophysiologies of the former responses are systemic inflammatory response syndrome (SIRS) and increased coagulation, which clinically manifest as tissue hypoxia/ischemia and multiple organ dysfunctions [3]. Adrie et al. [4] confirmed that successfully resuscitated cardiac arrest was followed by SIRS and activation of coagulation, both of which contributed to organ dysfunction, including the brain. Recent studies have indicated that disseminated intravascular coagulation (DIC) leads to organ dysfunction and affects the prognosis of out-of-hospital cardiac arrest (OHCA) patients [5, 6].

Inflammatory cytokine-initiated activation of tissue-factor-dependent coagulation, insufficient control of the anticoagulation pathways, and plasminogen activator inhibitor-1 (PAI-1)-mediated suppression of fibrinolysis characterize the pathogenesis of DIC [7]. From the first report of DIC following cardiac arrest [8], higher levels of tumor necrosis factor-α (TNF-α), interleukin-6 (IL-6) and IL-8 [4, 9, 10]; increased tissue factor levels [11]; insufficient levels of tissue factor pathway inhibitor (TFPI), antithrombin, protein C and protein S [4, 11]; and increased PAI-1 levels [12, 13] have been repeatedly confirmed during cardiopulmonary resuscitation (CPR) and after return of spontaneous circulation (ROSC). These changes lead to massive thrombin generation and consecutive fibrin formation [4, 6, 12]. Importantly, underlying conditions of DIC occasionally cause a simultaneous increase in fibrinolysis resulting from tissue-type plasminogen activator (t-PA), which is referred to as DIC with the fibrinolytic phenotype, as opposed to the thrombotic phenotype associated with elevated PAI-1 levels [14]. Both types of DIC have been recognized to affect the patient’s outcome [7, 14].

Marked increases in t-PA antigen and activity levels were followed by the PAI-1 expression during CPR and immediately after ROSC within 24 h [12]. In this study, therefore, significant imbalances between the levels of t-PA and PAI-1 during the first 24 h after cardiac arrest and resuscitation was noted which was coincided with the definition of DIC with the fibrinolytic phenotype.

According to the results of these previous studies on DIC, we investigated changes in coagulation and fibrinolysis markers during the first 24 h after OHCA and resuscitation. We tested the hypothesis that DIC with the fibrinolytic phenotype during the early phase of post-CPR is associated with SIRS, organ dysfunctions and affects the patient’s outcome.

## Methods

### Patient selection and data collection

From June 2000 to December 2011, all consecutive patients older than 12 years of age with successful ROSC from OHCA who were admitted to the ICU were eligible for this study. Patients who suffered to cardiac arrest due to trauma or burns, those on warfarin therapy, those with end-stage liver diseases, terminal illnesses or profound hypothermia, and those underwent percutaneous cardiopulmonary support (PCPS) were excluded. The Institutional Review Board of our institution approved this study and issued a waiver of informed consent.

A systematic review of the computer-based medical records of these patients was retrospectively conducted to provide baseline characteristics and DIC-related variables. Data regarding the platelet count, prothrombin time, prothrombin time ratio, fibrinogen, antithrombin, fibrin/fibrinogen degradation products (FDP), D-dimer and lactate were obtained at 4 time points within 24 h after successful ROSC: Time Point 01, immediately after ROSC to 4 h after ROSC; Time Point 02, 4 to 8 h after ROSC; Time Point 03, 8 to 16 h after ROSC; and Time Point 04, 16 to 24 h after ROSC. Day 0 data indicates the highest or lowest values of these 4 points measurements. Namely, the maximal worst values during this time period were considered for classfiyng patients with or without DIC and for determining the phenotype. The standard practice in our ICU for all patients is to draw blood several times a day in order to analyze the laboratory data including platelet count, coagulation and fibrinolysis. Blood gas analyses with lactate measurements were also frequently performed.

### Study setting and definitions

The levels of care provided by the emergency medical technicians (EMT) in our country are comparable to other advanced countries worldwide. The management of cardiac arrest was based on the 2000, 2005, and 2010 guidelines proposed by the International Liaison Committee on Resuscitation. Detailed pre-hospital care and CPR methods in our department can be found elsewhere [15].

Successful ROSC was defined as measurable blood pressure and pulse for more than 1 h and admission to the ICU, regardless of catecholamine use. Organ dysfunction was assessed by the Sequential Organ Failure Assessment (SOFA) score [16]. Multiple organ dysfunction syndrome (MODS) was defined as a SOFA score ≥ 12 [16]. The SIRS score was calculated according to the American College of Chest Physicians/Society of Critical Care Medicine consensus conference [17]. The diagnosis of DIC was made based on the Japanese Association for Acute Medicine (JAAM) DIC diagnosis criteria using day 0 data [18]. When the total score was ≥ 4, the DIC was established. The DIC phenotype was defined with reference to the criteria of Asakura, hyperfibrinolysis was defined as an FDP level of ≥ 100 μg/mL, and the FDP/D-dimer ratio was used as a surrogate marker of fibrin (ogen) olysis [14]. Tissue hypoperfusion was defined as a blood lactate level of ≥ 4 mmol/L based on the Surviving Sepsis Campaign Guidelines 2012 [19]. The outcome measure was the hospital all-cause mortality.

### Statistical analysis

Data are presented as the median and interquartile range. The IBM SPSS 22.0 for MAC OSX software program (IBM Japan, Tokyo) was used for the statistical analyses and calculations. Comparisons between the two groups were performed with the Mann-Whitney *U* test and either the Chi-square test or Fisher’s exact test when required. The relationships between the dependent and the independent variables were analyzed by a logistic regression analysis (the backward stepwise method based on likelihood) and the results were reported as the odds ratio and 95 % confidence intervals. The discriminatory performance for hospital death was evaluated using the area under the receiver operating characteristic (ROC) curve (AUC). Survival curves during hospital stay were derived according to the Kaplan-Meier methods and compared using the log-rank test. Differences with *p*-values <0.05 were considered to be statistically significant.

## Results

### Baseline patient characteristics

During the study period, a total of 1243 OHCA patients presented to our Emergency Department. After the exclusion of ineligible patients and patients with incomplete data for calculation of the DIC score, those performed PCPS were further excluded. Finally, 388 eligible patients were identified, who were divided into DIC (*n* = 208) and non-DIC patients (*n* = 180).

Table 1 shows the demographic data of the patients. Cardiac arrest due to a cardiac origin was less frequent in DIC patients. All DIC patients developed SIRS. DIC patients exhibited higher SIRS and SOFA scores associated with MODS, which associated with a significantly higher mortality than non-DIC patients (54.8 % vs. 23.9 %). Although some data were lacking (DIC, *n* = 182; Non DIC, *n* = 163), the Acute Physiology and Chronic Health Evaluation II scores of the DIC patients [34 (29–38)] were significantly higher than those of the non-DIC patients [29 (24–33)] (*P* < 0.001), suggesting that conditions of the DIC patients were more severe.

**Table 1:** Demographic and clinical characteristics of all patients

	Non DIC	DIC	*p* Value
	(180)	(208)	
Age (year)	66 (55–76)	71 (58–80)	0.020
Male sex (*n*,%)	112 (62.2)	124 (59.6)	0.604
Causes of cardiac arrest			
CNS/Cardiac/Respiratory/Asphyxia/Other/Unknown	13/99/24/32/11/1	18/86/30/37/32/5	–
Cardiac (*n*,%)	99 (55.0)	86 (41.3)	0.008
Initial rhythm			
VF/Asystole/PEA/Pulseless VT/Unknown	32/41/33/3/71	24/75/35/6/24	–
Shockable rhythm (*n*,%)	35 (19.4)	30 (14.4)	0.220
Witnessed arrest	75 (41.7)	92 (44.2)	0.681
Bystander CPR (*n*,%)	55 (30.6)	50 (24.0)	0.169
Shock by EMT (*n*,%)	42 (23.3)	44 (21.1)	0.626
Therapeutic hypothermia (*n*,%)	44 (24.4)	40 (19.2)	0.219
DIC score	2 (1–2)	5 (4–6)	0.000
SIRS score	3 (3–4)	4 (3–4)	0.048
SIRS (*n*,%)	178 (98.9)	208 (100)	0.215
SOFA day 0 score	6 (4–8)	9 (6–11)	0.000
MODS day 0 (*n*,%)	7 (3.9)	49 (23.6)	0.000
MODS day 5 (*n*,%)	10 (5.5)	67 (32.2)	0.000
Outcome death (*n*,%)	43 (23.9)	114 (54.8)	0.000

*CNS* central nervous system, *VF* ventricular fibrillation, *PEA* pulseless electrical activity, *VT* ventricular tachycardia, *CPR* cardiopulmonary resuscitation, *EMT* emergency medical technician, *DIC* disseminated intravascular coagulation, *APACHEII* Acute Physiology and Chronic Health Evaluation II, *SIRS* systemic inflammatory response syndrome, *SOFA* sequential organ failure assessment, *MODS* multiple organ dysfunction syndrome

### Serial changes in measured variables

**Fig. 1:**
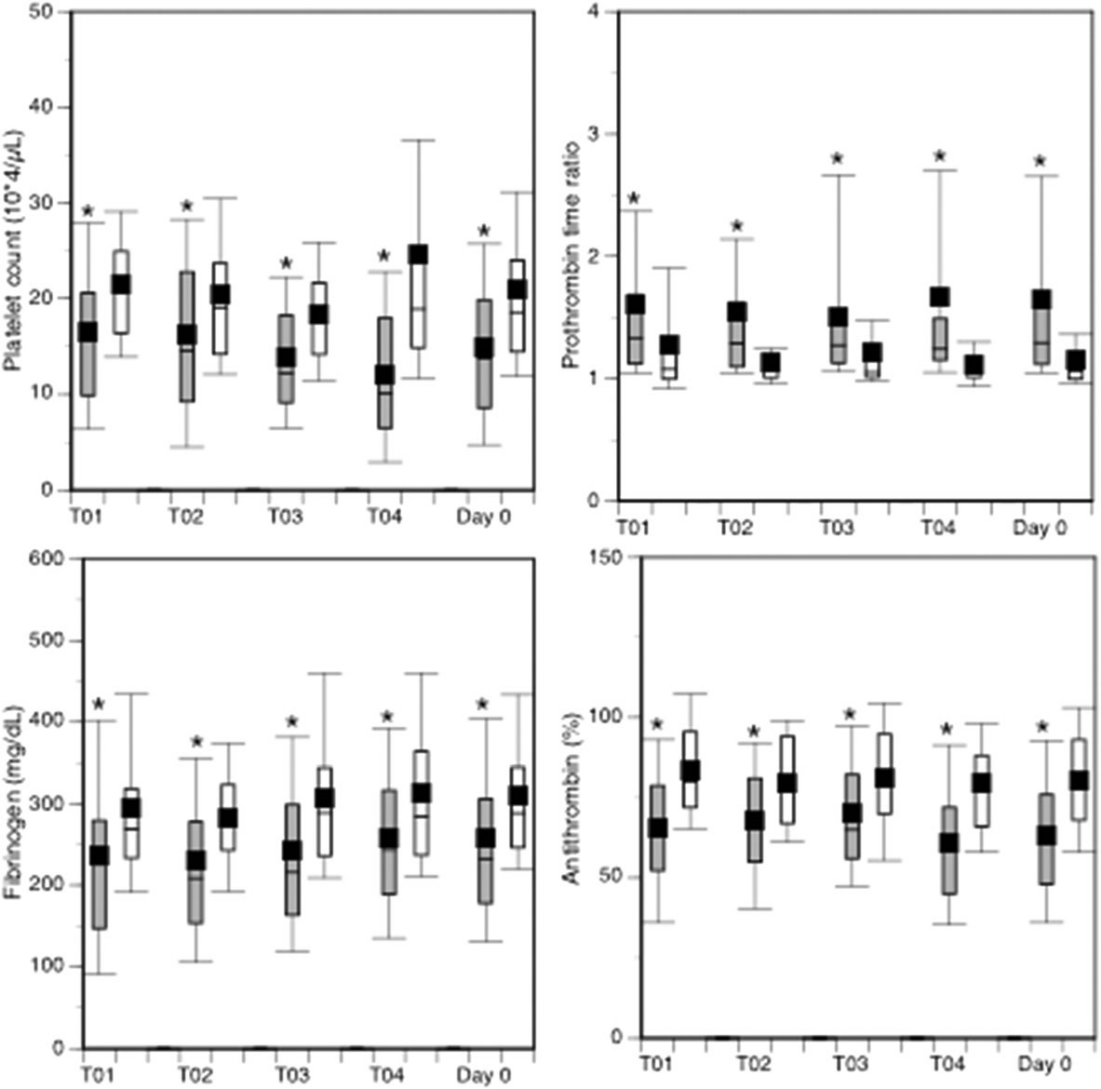
Box plots showing serial changes in the platelet counts, prothrombin time ratios, fibrinogen and antithrombin levels during the first 24 h in successfully resuscitated patients after OHCA. DIC patients (*grey* boxes) showed significantly lower platelet counts, more prolonged prothrombin time ratios, lower levels of fibrinogen and antithrombin than non-DIC patients (open boxes). Horizontal bars in the box indicate the median (*middle*) and interquartile ranges (*upper* 25 %, *lower* 75 %). Black squares in the box indicate the mean value. Top and bottom bars indicate the maximum and minimum values, respectively. *p < 0.001 vs. non-DIC patients

**Fig. 2:**
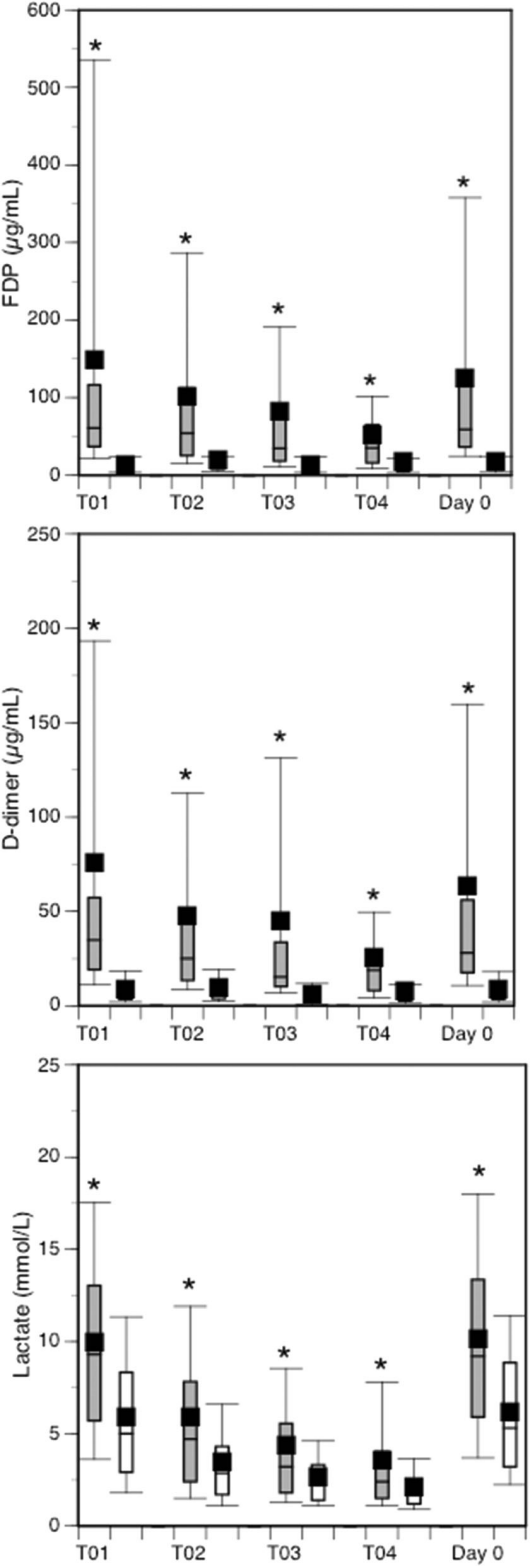
Box plots showing serial changes in FDP, D-dimer and lactate levels. DIC patients (*grey* boxes) showed significantly higher values of three variables than non-DIC patients (open boxes). Horizontal bars in the box indicate the median (*middle*) and interquartile ranges (*upper* 25 %, *lower* 75 %). Black squares in the box indicate the mean value. Top and bottom bars indicate the maximum and minimum values, respectively. *p < 0.001 vs. non-DIC patients

**Fig. 3:**
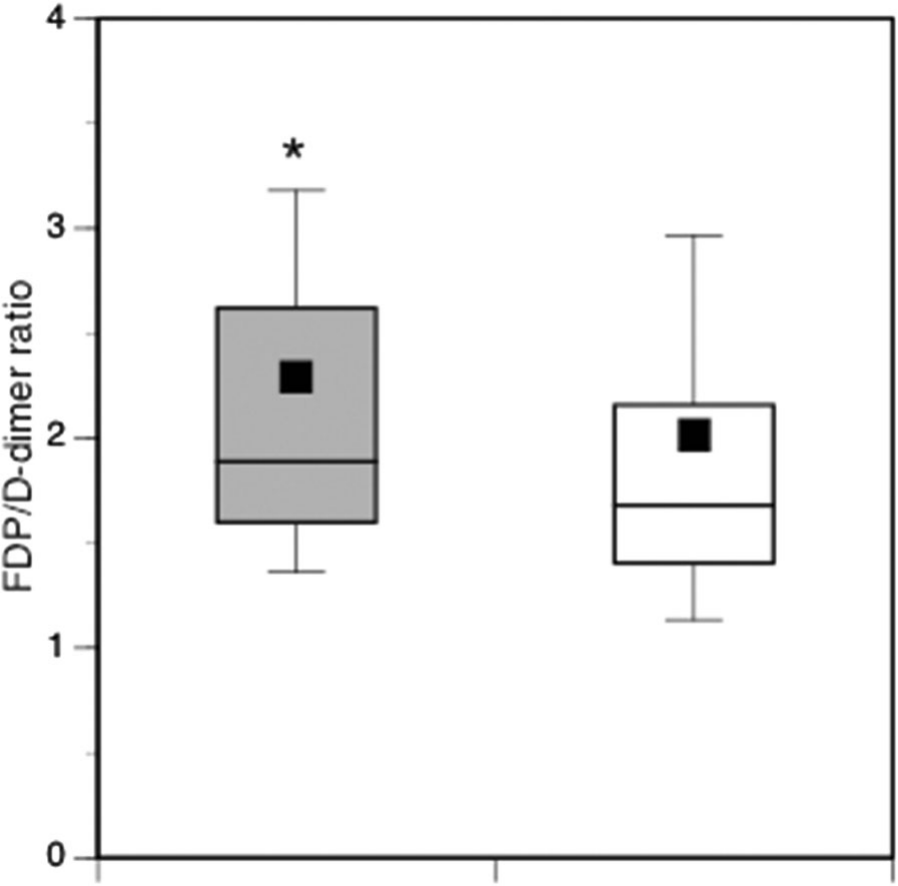
Box plots showing FDP/D-dimer ratios between DIC (*grey* boxes) and non-DIC patients (open boxes). DIC patients exhibited significantly higher ratios than non-DIC patients. Horizontal bars in the box indicate the median (*middle*) and interquartile ranges (*upper* 25 %, *lower* 75 %). *Black* squares in the box indicate the mean value. Top and bottom bars indicate the maximum and minimum values, respectively. *p < 0.001 vs. non-DIC patients

DIC patients continuously showed lower platelet counts, more prolonged prothrombin time ratios, and lower levels of fibrinogen and antithrombin than non-DIC patients (Fig. 1). Extremely high levels of FDP and D-dimer associated with marked increases in lactate levels were also observed in DIC patients (Fig. 2). In addition, FDP/D-dimer ratios in DIC patients were significantly higher than in non-DIC patients (Fig. 3). These results suggest consumption coagulopathy, insufficient anticoagulation, fibrin (ogen) olysis, and tissue hypoperfusion in DIC patients and that the DIC belongs to the fibrinolytic phenotype. The numbers of patients at each time point are provided in Additional file 1: Table S1.

### Subgroup analyses of DIC patients

DIC patients were subdivided into those with (*n* = 73) and without (*n* = 135) hyperfibrinolysis (Table 2). The markers of fibrinolysis and lactate levels of DIC patients with hyperfibrinolysis are presented in Table 3. Patients with hyperfibrinolysis had higher DIC and SOFA scores. Furthermore, MODS in these patients continued from day 0 to day 5, leading to a higher mortality rate of 68.5 % in comparison to patients without hyperfibrinolysis (41.7 %). The median FDP/D-dimer ratio of 2.0 in patients with hyperfibrinolysis was significantly higher than that in those without hyperfibrinolysis (Table 3). In addition, the lactate levels were significantly higher in those with hyperfibrinolysis than in those without fibrinolysis. These results suggest that DIC with hyperfibrinolysis is considered to be more severe DIC associated with extreme fibrin (ogen) olysis and tissue hypoperfusion, which results in worse outcome.

**Table 2:** Demographic and clinical characteristics of the DIC patients

	DIC		
	Hyperfibrinolysis No (135)	Hyperfibrinolysis Yes (73)	*p* Value
Age (year)	70 (58–79)	73 (59–82)	0.091
Male sex (*n*,%)	78 (57.8)	46 (63.0)	0.554
Causes of cardiac arrest			
Cardiac (*n*,%)	54 (40.0)	32 (43.8)	0.659
Initial rhythm			
Shockable rhythm (*n*,%)	22 (16.3)	8 (11.0)	0.408
Witnessed arrest	59 (43.7)	33 (45.2)	0.884
Bystander CPR (*n*,%)	28 (20.7)	22 (30.1)	0.173
Shock by EMT (*n*,%)	27 (20.0)	17 (23.3)	0.597
Therapeutic hypothermia (*n*,%)	30 (22.2)	10 (13.7)	0.146
DIC score	5 (4–5)	5 (4.5–6)	0.000
SIRS score	3 (3–4)	4 (3–4)	0.359
SIRS (*n*,%)	135 (100)	73 (100)	–
SOFA day 0 score	8 (6–11)	10 (7–13)	0.001
MODS day 0 (*n*,%)	23 (17.0)	26 (35.6)	0.004
MODS day 5 (*n*,%)	36 (26.7)	31 (42.5)	0.029
Outcome death (*n*,%)	64 (47.4)	50 (68.5)	0.004

*CNS* central nervous system, *VF* ventricular fibrillation, *PEA* pulseless electrical activity, *VT* ventricular tachycardia, *CPR* cardiopulmonary resuscitation, *EMT* emergency medical technician, *DIC* disseminated intravascular coagulation, *SIRS* systemic inflammatory response syndrome, *SOFA* sequential organ failure assessment, *MODS* multiple organ dysfunction syndrome

**Table 3:** Markers of fibrinolysis and lactate levels between DIC patients with and without hyperfibrinolysis

	Hyperfibrinolysis No (135)	Hyperfibrinolysis Yes (73)	*p* Value
FDP (μg/mL)	42.5 (28.2–57.6)	186.0 (110.3–404.5)	0.000
D-dimer (μg/mL)	20.3 (14.4–28.2)	74.3 (49.9–182.5)	0.000
FDP/D-dimer	1.8 (1.5–2.5)	2.0 (1.7–2.9)	0.007
Lactate (mmol/L)	8.1 (5.3–11.1)	11.3 (8.5–15.6)	0.000

Day 0 data are used for the FDP, D-dimer, and FDP/D-dimer values. The lactate data was obtained using data from time point 01

### Outcome analyses

**Fig. 4:**
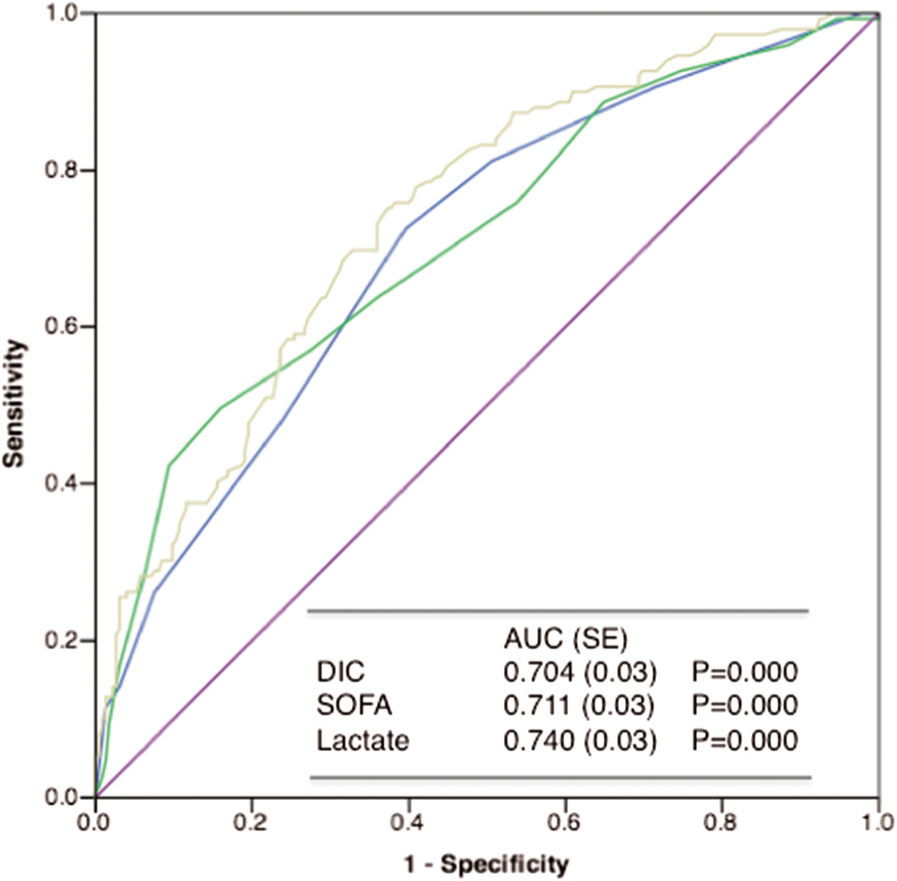
ROC curves of the DIC scores (*blue* line), SOFA scores (*green* line) and lactate levels (*yellow* line) for the prediction of hospital death of OHCA patients. All of these variables showed a good discriminative power to predict poor outcome of the patients. SE, standard error

**Fig. 5:**
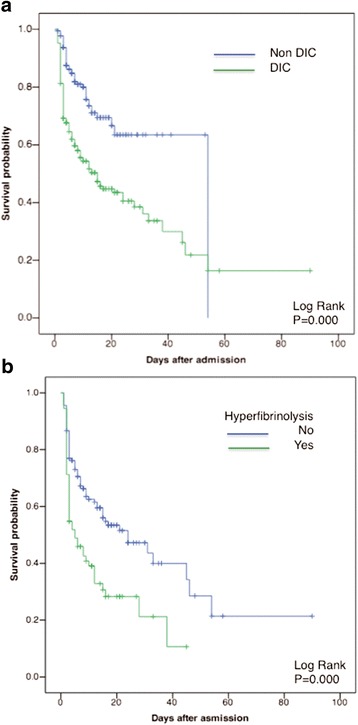
Kaplan-Meier curves showing the association between DIC (**a**) or hyperfibrinolysis (**b**) with hospital mortality. DIC and DIC with hyperfibrinolysis showed a significantly lower survival probability in the hospital than non-DIC patients and DIC without hyperfibrinolysis

Stepwise logistic regression analyses confirmed that DIC, SOFA scores, and lactate levels are independent predictors of patient death (Table 4). Hyperfibrinolysis also predicted patient death (Table 5). Table 5 shows that tissue hypoperfusion (as indicated by lactate level) is one of the causes of hyperfibrinolysis. ROC curves showed a significant discriminative performance of DIC and SOFA scores and lactate levels for patient death (Fig. 4). These results are important because the DIC score (score of one organ dysfunction [blood]) showed good discriminative power for the outcome compared with the SOFA score (score of multiple organ dysfunction). Kaplan-Meier curves showed that DIC, especially DIC with hyperfibrinolysis, significantly affected patient death (Fig. 5).

**Table 4:** Stepwise logistic regression analyses for prediction of the outcome (death)

	Odds ratio	*p* Value	95 % confidence interval
DIC score	1.171	0.041	1.006–1.364
SOFA score	1.178	0.001	1.073–1.292
Lactate	1.129	0.000	1.065–1.0197
Witnessed arrest	0.637	0.081	0.385–1.057
Cardiac origin	0.449	0.003	0.266–0.756
Shockable rhythm	0.400	0.024	0.180–0.887

The results of the final step of the analyses are shown. The dependent variables on the first steps: age, sex, DIC score (day 0), SOFA score (day 0), SIRS score (day 0), lactate level (time point 01), witnessed arrest, bystander CPR, shock by EMT, cardiac origin, and shockable rhythm

*DIC* disseminated intravascular coagulation, *SOFA* sequential organ failure assessment, *SIRS* systemic inflammatory response syndrome, *CPR* cardiopulmonary resuscitation, *EMT* emergency medical technician

**Table 5:** Logistic regression analyses for prediction of the outcome (death) and hyperfibrinolysis in DIC patients

	Odds ratio	*p* Value	95 % confidence interval
Outcome (enter method)			
SOFA score	1.204	0.000	1.094–1.324
Hyperfibrinolysis	1.938	0.038	1.036–3.626
Hyperfibrinolysis (stepwise method)			
Age	1.002	0.030	1.002–1.043
Bystander CPR	0.536	0.083	0.265–1.085
Lactate on time point 01	1.129	0.000	1.062–1.196

The stepwise method shows the results of the final step of the analyses. The dependent variables on the first steps: age, sex, lactate level, witnessed arrest, bystander CPR, shock by EMT, cardiac origin, and shockable rhythm

*DIC* disseminated intravascular coagulation, *SOFA* sequential organ failure assessment, *CPR* cardiopulmonary resuscitation, *EMT* emergency medical technician

## Discussion

The results of the present study demonstrate that OHCA patients with DIC during the early phase of post CPR are at risk of SIRS and MODS and that their condition is associated with a poor prognosis. The markedly higher FDP and D-dimer levels, and FDP/D-dimer ratio indicate that this type of DIC belongs to DIC with the fibrinolytic phenotype accompanied by fibrin (ogen) olysis. The outcomes of DIC patients with hyperfibrinolysis were worse than those observed in DIC patients without hyperfibrinolysis. The results of the logistic regression analyses suggest that tissue hypoperfusion, as evaluated by the lactate level, may be a cause of increased fibrinolysis.

Kim et al. [5] found that an increased initial DIC score in OHCA patients was an independent predictor for poor outcomes and early mortality risk. OHCA patients with a higher D-dimer level on admission had a poor outcome and the D-dimer levels were independent predictor of mortality [20]. Although hyperfibrinolysis was assessed by maximum lysis of rotational thromboelastometry, OHCA patients associated with hyperfibrinolysis showed a higher mortality rate, and hyperfibrinolysis was correlated with markers of tissue hypoperfusion including pH, base excess and lactate levels [21, 22]. Cardiac arrest due to asphyxia by drowning developed DIC with the fibrinolytic phenotype was associated with lower platelet counts and fibrinogen levels, prolonged prothrombin time, and significantly higher D-dimer levels, which led to a worse outcome [23]. The present study has importance in that it validates the previous studies, which separately confirmed the importance of DIC or increased fibrinolysis, and provides a unified concept of DIC with the fibrinolytic phenotype. It also reveals that this DIC phenotype is associated with an increased risk of mortality, especially those with hyperfibrinolysis.

For a long time, anoxia and endothelial injury were believed to be clearly established triggering stimuli for the appearance of circulating fibrinolytic activator and increased fibrinolysis [24–26]. Schneiderman et al. [27, 28] confirmed immediate increases in t-PA activity following arterial occlusion-induced ischemia both in humans and a rat model, which is attributable to the release of preexisting t-PA in Weibel-Palade bodies [29]. PAI-1 mRNA is initially expressed 4 h after hypoxia, followed by the appearance of PAI-1 antigen at 6 h, and reaches its peak levels at 20 to 24 h after hypoxia [30]. These chronological changes in the levels of t-PA and PAI-1 completely coincide with the changes in these variables during CPR and after ROSC in patients with OHCA [12]. These results suggest that hypoxia during pre-cardiac arrest, and ischemia/hypoxia during cardiac arrest and CPR result in whole body tissue hypoperfusion recognized as increased lactate levels, which is followed by increased fibrinolysis in DIC patients, as observed in the present and our previous study and asphyxia-induced OHCA patients [12, 23].

There are pre-cardiac arrest differences in the perfusion pressure and blood flow and the duration and degree of tissue hypoperfusion, which is dependent on the cause of cardiac arrests, such as shock, exsanguination, asphyxia, and ventricular fibrillation [1]. Ventricular fibrillation indicates sudden onset of ischemia without pre-cardiac arrest hypoxia. Therefore, in the present study, a low incidence of cardiac arrest due to a presumed cardiac origin in DIC patients might, in part, explain the finding that causes of cardiac arrest influence the degree of fibrinolysis. However, a subgroup analysis of a previous study confirmed that the magnitude of increased risk of a DIC-dependent poor prognosis in cardiac etiology was similar to that of the overall study population [5]. Many studies have reported that the time to first basic life support and duration of CPR were two of the main causes of hyperfibrinolysis [5, 21–23]. Although these parameters were not obtained in the present study, low odds ratio of bystander CPR in Table 5 indirectly suggests that a shortened ischemia period due to bystander CPR may reduce the risk of hyperfibrinolysis. Lastly, evenly distributed therapeutic hypothermia excluded the effects of hypothermia on DIC and increased fibrinolysis in the present study. Taken together, these results suggest that the duration of hypoxia/ischemia, but not etiologies of cardiac arrest, may be a primary determinant of the development of DIC with the fibrinolytic phenotype in OHCA patients.

Activated protein C did not increase in OHCA patients [22]. Another study confirmed that activated protein C levels are not high enough to inhibit PAI-1 [23]. Neither syndecan-1 nor endothelial heparin sulfate levels were elevated in DIC with the fibrinolytic phenotype observed in asphyxia-induced OHCA [23]. Furthermore, other heparinase-sensitive glycosaminoglycans were not elevated [23]. These results suggest that participations of activated protein C and phenomena referred to as auto-heparinization are highly unlikely during hyperfibrinolysis in patients with OHCA.

Limitations. The results of the present study is based on retrospective analyses of OHCA in a single center and limited by an incomplete data set. Although increased fibrinolysis can be confirmed, the supposed causes of this phenomenon, such as t-PA and PAI-1, were not measured. Bleeding or a bleeding tendency, and the time to ROSC, and outcome scores such as cerebral performance category were not evaluated due to a lack of data. However, the magnitude of the results of this study suggests that a prospective multicenter study is needed to evaluate the effect of DIC on the outcome of OHCA patients.

## Conclusions

In OHCA patients, DIC during the early phase of post-CPR exhibited lower platelet counts, consumption coagulopathy, and insufficient antithrombin levels associated with increased fibrin (ogen) olysis assessed by higher FDP/D-dimer ratios, which can explain DIC of the fibrinolytic phenotype. DIC patients, especially those with hyperfibrinolysis, more gives rise to MODS associated with SIRS, leading to worse outcomes than those without DIC. Tissue hypoperfusion due to hypoxia and ischemia during cardiac arrest and CPR may be considered to be one of the causes of increased fibrinolysis in this type of DIC. The present study has validated previous studies that separately confirmed existence of DIC or increased fibrinolysis during the early post-CPR phase in OHCA patients. This study has further importance in that it unifies DIC and increased fibrinolysis as a single condition in the fibrinolytic phenotype of DIC and it recognized that this phenotype is associated with poor outcome.

## Additional file

Additional file 1: Table S1. Numbers of the patients. (DOCX 48 kb)

## Abbreviations

CNS: Central nervous system
CPR: Cardiopulmonary resuscitation
DIC: Disseminated intravascular coagulation
EMT: Emergency medical technician
FDP: Fibrin/fibrinogen degradation products
MODS: Multiple organ dysfunction syndrome
OHCA: Out-of-hospital cardiac arrest
PCPS: Percutaneous cardiopulmonary support
PEA: Pulseless electrical activity
ROSC: Return of spontaneous circulation
SIRS: Systemic inflammatory response syndrome
SOFA: Sequential organ failure assessment
VF: Ventricular fibrillation
VT: Ventricular tachycardia
