# Supplementary material for: Disseminated intravascular coagulation with the fibrinolytic phenotype predicts the outcome of patients with out-of-hospital cardiac arrest
Source: Thromb J. 2016 Sep 21;14:43. doi: 10.1186/s12959-016-0116-y (PMC5030731; doi:10.1186/s12959-016-0116-y)
Supplement: Additional file 1: Table S1. — Numbers of the patients. (DOCX 48 kb) [file 12959_2016_116_MOESM1_ESM.docx]

| Supplementary Table 1 Numbers of the patients | | | |  |  |
| --- | --- | --- | --- | --- | --- |
|  | Time point 01 | Time point 02 | Time point 03 | Time point 04 | Day 0 |
| Platelet count | 185 | 173 | 152 | 95 | 388 |
| Prothrombin time ratio | 172 | 167 | 148 | 95 | 388 |
| Fibrinogen | 172 | 164 | 147 | 94 | 388 |
| Antithrombin | 152 | 138 | 128 | 78 | 388 |
| FDP | 162 | 157 | 136 | 89 | 388 |
| D-dimer | 163 | 158 | 138 | 88 | 388 |
| Lactate | 374 | 351 | 342 | 327 | 388 |
